# Turkish translation, cross-cultural adaptation and reliability of the Groningen Frailty Indicator

**DOI:** 10.1186/s12877-023-04445-5

**Published:** 2023-11-17

**Authors:** Merve Güner, Serdar Ceylan, Arzu Okyar Baş, Meltem Koca, Burcu Balam Doğu, Meltem Gülhan Halil, Mustafa Cankurtaran, Cafer Balcı

**Affiliations:** Faculty of Medicine, Department of Internal Medicine, Division of Geriatric Medicine, Hacettepe University, 06230 Sıhhıye, Ankara, Türkiye

**Keywords:** Frailty, Frailty tools, Physical phenotype, Groningen Frailty Indicator

## Abstract

**Background:**

Frailty is an important geriatric syndrome that can be seen as a way of recognizing and distinguishing the complex health conditions of older people. Due to the time limitation, short and simple instruments are most feasible in clinical practice, and several quick screening tools have been developed and validated, Groningen frailty indicator (GFI) is one of these scales. We aimed to validate and evaluate the reliability of the GFI in outpatient older adults in the Turkish population.

**Methods:**

A total of 101 older patients were enrolled to the study. GFI was scored by a geriatrician for every patient at first admission to the geriatric outpatient clinic. Fried Physical Frailty Phenotype (FPFP) was performed as a reference test.

**Results:**

The median age (IQR) was 72.0 (10.0) and 62.4% of the study population (n = 63) was female. Based on the GFI, 34 patients (33.7%) were defined as robust, and 67 patients (66.3%) were defined as living with frailty. There was a statistically significant concordance between GFI and FPFP (Cohen’s kappa: 0.415 p < 0.001). GFI had excellent consistency in inter-rater reliability (Cronbach’s alpha: 0.99, 95% CI 0.97-1.00) and in intra-rater reliability (Cronbach’s alpha: 0.99, 95% CI 0.96-1.0).

**Conclusion:**

Our study showed that GFI is a valid and reliable scale in the Turkish older population.

**Supplementary Information:**

The online version contains supplementary material available at 10.1186/s12877-023-04445-5.

## Introduction

Frailty is an important geriatric syndrome that can be seen as a way of recognizing and distinguishing the complex health conditions of older people [1]. A simple way of viewing frailty is the idea that minor stressors can cause major functional and physical decline with associated adverse outcomes. However, there is no single accepted definition of frailty. As the definitions for frailty are not standardized, the reported prevalence of the frailty syndrome ranges from 4.0 to 59.1% [2]. Although there is no gold standard method for the identification of frailty, the usual and best method is still the comprehensive geriatric assessment (CGA) [3]. CGA requires the evaluation of physical, cognitive, affective, social, financial, and environmental components. Owing to the time limitation short and simple instruments are most feasible in clinical practice, and several quick screening tools have been developed and validated. The two most well-known and accepted concepts of frailty are the Physical Frailty Phenotype and the Cumulative Deficit Model.

Physical frailty is a medical syndrome with multiple causes and contributors and is characterized by diminished strength, endurance, and reduced physiologic function that increases an individual’s vulnerability to developing increased dependency and/or death [4]. Physical frailty is more complicated than sarcopenia alone and differs from multimorbidity. The cumulative deficit model suggests that the accumulation of potential health deficits leads to frailty, including diseases and disabilities. Despite its high predictive validity, this method is rarely utilized in clinical situations in being time-consuming [5]. There are numerous tools developed to screen and identify frailty in community-dwelling older adults including Tilburg Frailty Indicator, Edmonton Frailty Scale, FRAIL index, and Fried’s Frailty Phenotype [6]. Most of these instruments focus on malnutrition and disability, however, some instruments consider frailty as a multidimensional syndrome. Groningen frailty indicator (GFI) is one of the scales that evaluates frailty as physical condition, cognition, social, and psychological aspects [7].

GFI was developed by Steverink et al. at 2001 [7]. GFI assessed frailty through 4 different dimensions, including physical status, cognition, social status, and psychological status. GFI defines frailty from the self-reported 4 domains (Physical status, cognition, social status, and psychological status) including 15 items. So a total score of 4 or more can be caused by many different aspects of frailty-related conditions that managing the frailty could be more specific. GFI is a valid and reliable tool for the recognition of frailty in both community-dwelling older adults and institutionalized older adults [8, 9].

Despite disagreement on the best methodology to identify frailty in older adults, there has been an emerging trend toward the recognition of the potential importance of screening for frailty to assist in general decision-making [10]. Since GFI implements multidomain evaluation, it stands out one step further among all other tools, and so using the GFI properly is essential for identifying frailty. Herein, we aimed to validate and evaluate the reliability of the GFI in outpatient older adults, which was developed to screen frailty in the Turkish population.

## Material and method

### Study population

A total of one hundred and one patients aged 65 years and older who were admitted to the Hacettepe University Hospital outpatient clinic of geriatric medicine were enrolled in the study. The patients with acute illnesses, delirium, and who had failed to give informed consent were excluded from the study. The CGA was performed on all participants. Demographic data (age, sex, education, and marital status) and comorbidities were recorded from the medical records or gathered through statements of patients or their caregivers. Geriatric syndromes including falls, osteoporosis, urinary incontinence, and polypharmacy were also determined. Those who have a history of a fall within one year before admission to the outpatient clinic were accepted as having a positive fall history and polypharmacy was defined as the usage of 5 or more medications [11]. Multimorbidity was defined as the presence of two or more chronic conditions.

### Comprehensive geriatric assessment

The gold standard method for the evaluation of frailty is accepted as the CGA. CGA is a comprehensive, multidisciplinary approach assessing the physical, functional, cognitive, psychosocial, and medical aspects of frailty in older adults. CGA was performed using standardized tools including Mini-Mental State Examination (MMSE), Mini Nutritional Assessment short form (MNA-SF), Yesavage’s Geriatric Depression Scale (GDS), Katz Activities of Daily Living (ADL) scale, and Lawton Brody Instrumental Activities of Daily Living (IADL). The functionality of the patient was scored by Katz ADL [12] (0–6 points) evaluating the bathing, dressing, feeding, using the toilet, transferring and continence and IADL (0–8 points) was scored [13, 14] according to independency in using the telephone, shopping, preparing the food, doing laundry, housekeeping, transporting, using the medications and handling the finance. Nutritional status was investigated with MNA-SF (0–14 points) [15]. MMSE and GDS were performed for the evaluation of cognitive functions and depressive symptoms, respectively [16, 17]. For the screening of sarcopenia, the SARC-F questionnaire was conducted [18]. Muscle strength was assessed by handgrip strength (HGS) measured by a dynamometer (Takei TKK 5401 Digital Handgrip Dynamometer, Niigata-City, Japan). Grip strength was measured in an upright position with the arms parallel to the body. The participants were asked to apply the maximum grip strength 3 times with the unsupported dominant hand [19]. The highest value of the three trials was recorded as the HGS. Low HGS was defined as HGS < 16 kg and < 27 kg, for women and men, respectively [18]. Gait speed was assessed by a 4-m walking test. Participants were informed to walk over a 4-m course at their usual speed. The cut-off point was accepted as < 0.8 m/Sect. [20].

### Assessment of frailty

#### Groningen frailty indicator

The Groningen Frailty Indicator (GFI) is a widely used tool to assess frailty in older adults. The GFI is a multidimensional assessment tool that takes into account various domains of health and functioning to provide a comprehensive understanding of an individual’s frailty status. The GFI comprises 15 items organized into four domains of frailty: The Physical Domain (Unintentional weight loss, mobility problems, balance issues, vision problems, and hearing problems), the Cognitive Domain (Memory problems, and concentration problems), the Psychological Domain (Depressive symptoms, and anxiety symptoms), and the Social Domain (Loneliness, limited social relationships, and financial difficulties).

Each of the 15 items in the GFI is scored as either “0” (absence of the problem) or “1” (presence of the problem). The scores are then summed to create a total GFI score, which can range from 0 to 15. A higher GFI score indicates a higher level of frailty. Typically, a cutoff score is used to classify individuals into different frailty categories: non-frail/ robust (GFI score 0–3) and living with frailty (GFI score 4 or higher).

The GFI is usually administered by a trained geriatrician. To assess the intra-rater reliability, the same geriatrician performed the revised GFI evaluation two weeks later on the same 15 patients. For inter-rater reliability, a second geriatrician performed the GFI among ten random patients during their initial visit in a different examination room.

#### Fried physical frailty phenotype

Fried Physical Frailty Phenotype (FPFP) contains five criteria including weight loss, burnout, loss of strength, limitation in physical activity, and slow walking speed. *Weight loss* was defined as a loss of 4.5 kg or more involuntarily in the last year. *Burnout* was questioned by ‘Have you ever felt unwilling or unable to do most of your work in the last week?’. *Loss of strength* was measured by handgrip strength of < 16 kg for women and < 27 kg for men. *Limitation in physical activity* was asked by ‘Have you gone outside less than once a week in the last 1 year?’. *Slow walking speed* is determined by < 0.8 m/sec. The presence of three or more of the five criteria was considered as ‘living with frailty’, with one or two criteria as ‘living with pre-frailty’, with none of the criteria as ‘robust’. Patients living with frailty and pre-frailty were analyzed altogether.

### Translation

To develop the Turkish version of the revised GFI, we obtained copyright permission from the instrument’s developer. To ensure linguistic equivalence, we conducted a forward translation of the instrument from English to Turkish. The English-to-Turkish translation of the GFI was carried out by two native Turkish-speaking physicians proficient in English. An expert panel of clinicians convened to review and compare the forward translations, identifying discrepancies and addressing language and cultural context.

Following the expert panel review, a synthesized Turkish version of the instrument was developed. After the agreement of all the authors on the Turkish version, backward translation to English was operated by two native English speakers who were blinded to the original scale. This step served to verify content equivalence between the synthesized Turkish version and the English version. A preliminary version of the adapted test in Turkish was administered to a sample of the target population (N = 10). This sample size was determined to ensure sufficient feedback for item evaluation. The sample size was calculated using two rater kappa statistics [21] by providing 90% power to determine the correct kappa. Data were collected to assess the reliability (internal consistency, inter/intra-rater reliability) and validity (construct validity, criterion validity) of the adapted screening instrument. The scale was administered to outpatient older adults to assess cultural adaptation as a final step of language validation.

### Statistical analysis

Statistical analysis was performed using SPSS 24.0. The sample size was calculated using two rater kappa statistics [21] by providing 90% power to determine the correct kappa. Robust and pre-frail/frail frequencies were used for sample size calculation, which were 0.42 and 0.58 respectively [22]. The categorical variables were presented as numbers and percentages. Variables were examined using visual and analytical methods to determine whether they were normally distributed. Variables were presented as means and standard deviation or median and interquartile range (IQR) concerning normal distributions. To evaluate the construct validity of the GFI, Cohen’s Kappa coefficient was calculated accepting the FPFP scale as the reference tool. Inter-rater and intra-rater reliability of the GFI were evaluated by Cronbach’s Alpha. Sensitivity, selectivity, and positive and negative predictive values were calculated. Correlation analyses were performed with a Pearson or Spearman correlation test based on the distributions of the variables. P < 0.05 was considered statistically significant.

Receiver operating characteristics (ROC) curve analysis was performed to estimate the capacity of GFI to predict frailty status. The sensitivity, specificity, and positive and negative predictive values were presented when a significant cutoff value was present with 95% confidence interval (CI) and a 5% level of significance (P < 0.05).

### Ethical statement

The study protocol was conducted following the Declaration of Helsinki and it was approved by the local ethics committee of Hacettepe University. Informed consent was provided by all participants or their legal guardians after providing verbal and written information about the study.

## Results

One hundred and one (101) patients were included in the study. The median age (IQR) was 72.0 [10] and 62.4% of the study population(n = 63) was female. According to the Fried Physical Frailty Phenotype, 69 patients (68.3%) were found to be living with frailty and 32 patients (31.7%) were defined as robust. Based on the GFI, 34 patients (33.7%) were defined as robust, and 67 patients (66.3%) were defined as living with frailty. In the entire study group, 70 (69.3%) patients had multimorbidity. The most common chronic diseases were hypertension (70.3%), diabetes mellitus (49.5%), and coronary artery disease (17.8%), respectively. The characteristics and demographic features of the study population were given in Table 1.

**Table 1:** The characteristic and demographic features of the Study Population

	Study Population (N = 101)
**Demographics**	
**Age, years**	72.0 [10.0]
**Sex, female**	63 (62.4)
**Illiterate**	23 (22.8)
**Marital Status, married**	63 (62.4)
**BMI, kg/m** ^**2**^	30.12 ± 5.74
**Smoking**	37 (36.6)
**Chronic Diseases**	
**Diabetes Mellitus**	50 (49.5)
**Hypertension**	71 (70.3)
**Coronary Artery Disease**	18 (17.8)
**Congestive Heart Failure**	6 (5.9)
**Atrial Fibrillation**	12 (11.9)
**Cerebrovascular Event**	10 (9.9)
**Chronic Kidney Disease**	4 (4.0)
**Chronic Obstructive Pulmonary Disease-Asthma**	9 (8.9)
**Malignancy**	10 (9.9)
**Hypothyroidism**	10 (9.9)
**Multimorbidity (≥ 2 chronic diseases)**	70 (69.3)
**Comprehensive Geriatric Assessment**	
**Dementia**	4 (4.0)
**Depression**	31 (30.7)
**Osteoporosis**	24 (23.8)
**Falls**	22 (21.8)
**Polypharmacy**	53 (52.5)
**Drug number**	5.0 [4.0]
**Urinary Incontinence**	42 (41.6)
**Katz Index of Independence in ADL**	6.0 [1.0]
**Lawton-Brody Instrumental ADL**	8.0 [1.0]
**Mini Nutritional Assessment-Short Form**	13.0 [4.0]
**Mini-mental State Exam**	28.0 [4.8]
**Yesavage Geriatric Depression Scale**	2.0 [6.0]
**SARC-F**	1.0 [3.0]
**Grip strength, kg** **Females** **Males**	17.75 ± 4.94 27.39 ± 7.41
**Gait speed, m/sec**	0.94 ± 0.35
**Fried Physical Frailty Phenotype**	1.0 [3.0]

Variables were presented as n(%), median [Interquartile range] or mean ± standard deviation

BMI; Body mass index, ADL;Activities of daily living

Comprehensive geriatric assessment was performed on the study group and geriatric syndromes were questioned. Median Katz ADL was 6.0 (1.0), and median Lawton-Brody IADL was 8.0 (1.0). The median MNA-SF score was 13.0 (4.0), the median of MMSE was 28.0 (4.8), and the median of Yesavage GDS was 2.0 (6.0). Among the variables related to sarcopenia, the median of SARC-F was 1.0 (3.0), the mean gait speed was detected as 0.94 ± 0.35, and the mean handgrip strength was 17.75 ± 4.94 kg for females and 27.39 ± 7.41 kg for males. The most frequently detected geriatric syndrome was polypharmacy (52.5%), followed by urinary incontinence (41.6%). Four patients (4.0%) have been diagnosed with dementia, and 31 patients (30.7%) had depression.

There was a statistically significant concordance between GFI and FPFP (Cohen’s kappa: 0.415 p < 0.001). GFI had excellent consistency in inter-rater reliability (Cronbach’s alpha: 0.99, 95% CI 0.97-1.00) and in intra-rater reliability (Cronbach’s alpha: 0.99, 95% CI 0.96-1.0) (Table 2). Inter-rater and intra-rater reliabilities of each item in the GFI were also investigated and presented in Table 2. There was excellent inter-rater reliability on walking around outside, dressing and undressing, going to the toilet, and prescription medications. There was very good inter-rater reliability on physical fitness, having problems due to poor vision and hearing, weight loss, memory, and social and psychological domains. Intra-rater reliability was excellent in all items, except for having problems due to poor vision (0.89) and being nervous and downhearted (0.79). The sensitivity of the GFI determined according to the FPFP scale was 79.71%, whereas the specificity was 62.5%, the positive predictive value and the negative predictive value were 82.09% and 58.82%, respectively. The positive and negative likelihood ratios were calculated as 2.13 and 0.32, respectively (Table 3). There was a significant correlation between GFI and FPFP (Spearman rho coefficient: 0.66, p < 0.001).

**Table 2:** Groningen frailty indicator and reference test concordance results

		Groningen Frailty Indicator		Kappa	Approximate Significance
		Robust	Frail		
**FPFP**	**Robust**	20 (58.8)	12 (17.9)	0.415	< 0.001
	**Pre-frail/Frail**	14 (41.2)	55 (82.1)		
**Inter-rater reliability**		-	-	0.783	0.01
**Intra-rater reliability**		-	-	1.0	< 0.001

FPFP; Fried Physical Frailty Phenotype

**Table 3:** The inter-rater and intra-rater reliability of every item and total score of Groningen frailty indicator

	Inter-rater Reliability	Intra-rater Reliability
	Cronbach’s alpha	Cronbach’s alpha
*Physical Domain*		
Shopping	0.78	1.0
Walking around outside	1.0	1.0
Dressing and undressing	1.0	1.0
Going to the Toilet	1.0	1.0
Mark the physical fitness	0.76	0.98
Poor Vision	0.75	0.89
Poor Hearing	0.85	1.0
Weight Loss	0.86	1.0
Prescription of 4 or more medicines	0.90	0.90
*Cognitive Domain*		
Cognition	0.80	0.79
*Social Domain*		
Part of social network	0.85	0.95
Attention other people	0.88	0.91
Help other people	0.86	0.90
*Psychological Domain*		
Calm and Relaxed	0.76	0.98
Nervous and Downhearted	0.83	0.79
*Total Score*	0.99(0.97-1.0)	0.99(0.96-1.0)

The study population was divided into two categories according to their frailty status by GFI and the prevalence of the geriatric syndromes was investigated. No difference was observed in terms of multimorbidity(p = 0.11) and fall history(p = 0.08). However urinary incontinence and polypharmacy were more common in patients living with frailty according to GFI (50.7% vs. 23.5%, p = 0.01 and 59.7% vs. 38.2%, p = 0.04, respectively). The patients living with frailty were more dependent on basic and instrumental activities of daily living than robust patients (p = 0.001 and p = 0.001, respectively). Lower scores in MNA-SF and MMSE and higher scores in GDS and SARC-F questionnaires were also observed in patients living with frailty. The handgrip strength was lower and gait speed was slower in the patients living with frailty defined by GFI (p = 0.001 and p = 0.02). Detailed results are shown in supplementary Table 1.

**Fig. 1:**
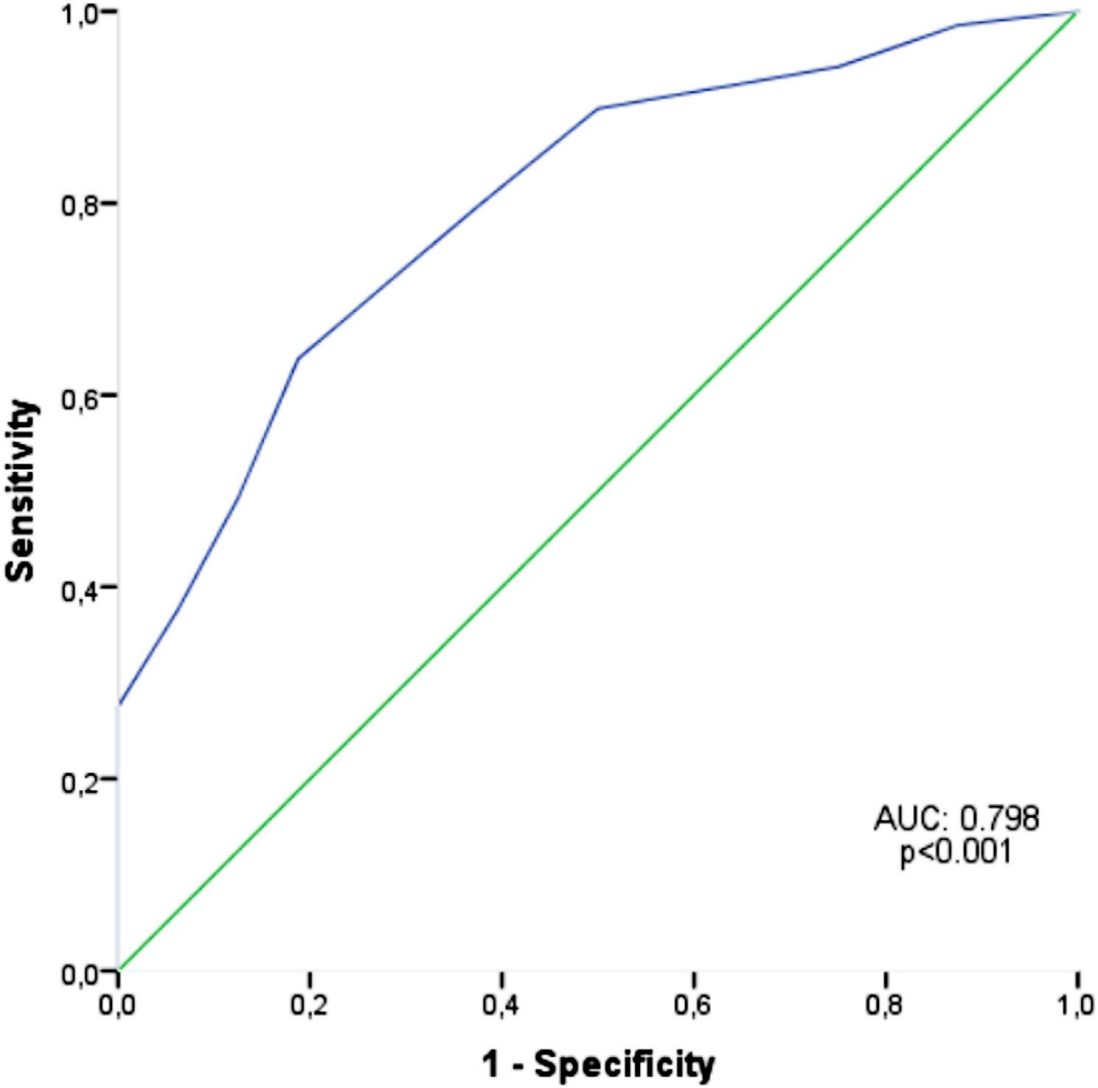
ROC curves of GFI to predict frailty status. ROC, receiver operating characteristic, AUC,area under curve

ROC curves to predict frailty are shown in Fig. 1. The thresholds were calculated separately for GFI scores ≥ 4 and ≥ 5 for the prediction of living with frailty prediction. AUC was calculated at 0.798, sensitivity and specificity were 79.7% and 63.5%, and PPV and NPV were found 82.09 and 58.82, respectively for threshold 4. Sensitivity and specificity were found 63.8% and 81.2%, respectively for threshold 5 and PPV and NPV were 88.0 and 50.98, respectively.

The correlations between the total score of GFI and comprehensive geriatric assessment parameters were shown in Table 4. Functional status defined by Katz ADL and Lawton-Brody IADL, nutritional status evaluated by MNA-SF, and psychological status assessed by Yesavage GDS, SARC-F, HGS, EAT-10, and the number of medications were all significantly correlated with the GFI.

**Table 4:** Correlations between GFI and other comprehensive geriatric assessment parameters

	Groningen Frailty Indicator	
	r	p-value
**KATZ ADL**	-0.388	< 0.001
**Lawton-Brody IADL**	-0.482	< 0.001
**Handgrip Strength, kg**	-0.502	< 0.001
**SARC-F**	0.557	< 0.001
**EAT-10**	0.226	0.023
**Yesavage GDS**	0.658	< 0.001
**MMSE**	-0.425	< 0.001
**MNA-SF**	-0.517	< 0.001
**Number of medications**	0.423	< 0.001

ADL; Activities of daily living, IADL;Instrumental Activities of Daily Living, GDS; Geriatric Depression Scale, MMSE; Mini-mental State Examination, MNA-SF; Mini- nutritional Assessment short form

## Discussion

The foremost purpose of our study is to determine the validation and reliability of the GFI in the Turkish older population. It was shown that GFI has a good concordance with the FPFP scale and is an appropriate tool for detecting frailty among the Turkish older population. It is an easy-to-apply and rapid tool for determining frailty, and the inter-rater and intra-rater reliability study among geriatric outpatient adults was also strong.

GFI could be used to identify frailty in both community-dwelling and institutionalized older adults [8, 9]. In a study from China conducted on nursing home residents, the Physical frailty phenotype was used as a reference tool. It was stated that GFI had good internal consistency (ICC = 0.712) and an excellent test-retest reliability [23]. It was also demonstrated that GFI had good internal consistency (Cronbach’s alpha = 0.72) in Turkish nursing home residents [24]. On the other hand, our study is different from the aforementioned studies since the study population is outpatient older adults. Residents of nursing homes typically have higher care needs and often suffer from multiple chronic health conditions, often have limited autonomy due to their medical conditions and the need for constant care and their social and environmental dynamics in nursing homes are distinct, characterized by a shared institutional setting. In contrast, older adults in community settings tend to be more independent and have varying levels of care needs. Therefore, it is essential to tailor assessment methods to each group’s unique characteristics and requirements to ensure that the assessments accurately reflect their specific needs and challenges. According to our findings, GFI has good reliability and consistency in community-dwelling older Turkish adults.

The GFI is shown a range of Cronbach’s alpha values, from 0.68 to 0.73, indicating moderate internal consistency in the previous studies [8, 9]. Recently, validity and reliability studies of the GFI in other languages have also been published. The German translation of GFI was also valid with 100% sensitivity and 80% specificity according to the physical frailty phenotype [25]. Furthermore, the Chinese version of the GFI was strongly correlated with the Frailty Index, which is different from our study using the FPFP scale for validation. The Chinese version of GFI has also good internal consistency (Cronbach’s alpha = 0.64) [26]. The Romanian version of GFI is a feasible and valid instrument to assess frailty (Cronbach’s alpha = 0.746) [27]. The validation of GFI in different languages helps to contribute of the evaluation of frailty with multidimensional approach, addressing not only physical issues but also psychological and social aspects that may contribute to frailty. GFI could provide a holistic point of view of the patient’s well-being, leading to more personalized and targeted interventions.

The prevalence of frailty varies depending on the specific components assessed, as observed in our study. In our investigation, the observed frailty prevalence was 66.3%, notably higher than the reported rates of 12–24% in previous research [28]. Notably, it is important to acknowledge that the GFI lacks a designated level for prefrailty. In studies employing physical frailty criteria, the prefrailty prevalence was identified at 46%, while studies utilizing the deficit accumulation model reported a prefrailty prevalence of 49% [28]. In alignment with these findings, another study documented a frailty prevalence of 60% based on the GFI, which closely mirrors our own results [9]. Consequently, our analysis suggests that the GFI exhibits a high degree of predictability and reliability.

It has been demonstrated that frailty is closely linked to adverse health outcomes, including early mortality, hospitalization, and institutionalization. A systematic review has identified three distinct types of frailty assessment instruments: those with a physical focus, multidomain focus, and the deficit accumulation method [29]. Consistent with the findings of this review, frailty, as assessed by all three types of instruments, has been associated with increased mortality, loss of activities of daily living, hospitalization, physical limitations, falls, and fractures [29]. As the prevalence and severity of frailty increase, there is a concomitant rise in health expenditures and healthcare utilization across inpatient, post-acute, and outpatient care sectors [30]. In a study that compared three different frailty scales (GFI, Tilburg Frailty Indicator, and Sherbrook’s Postal Questionnaire) with regard to the development of disability, mortality, and hospital admission, it was found that 38% of patients living with frailty, as defined by GFI, experienced functional decline within one year. Moreover, during a one-year period, 20% of these frail individuals were admitted to the hospital, and the mortality rate was 4% [31]. In another study examining the predictive performance of four frailty screening tools among community-dwelling older adults, individuals identified as frail by GFI faced a 3.8-fold increased risk of disability, a 3.4-fold increased risk of institutionalization, and a 1.9-fold increased risk of mortality [32]. Consequently, GFI holds promise as a valuable tool for assessing the risk of loss of independence, institutionalization, and mortality. However, it is important to acknowledge a limitation of our study—namely, the absence of long-term data. Future research should consider incorporating long-term follow-up data to enhance our understanding of the enduring impact of frailty on health outcomes.

Considering the frequency of frailty, its relationship with adverse health outcomes, and its cost to the health system, early recognition of frailty is important in terms of reversing the frailty in its early stages, preventing the negative consequences of this geriatric syndrome, and providing individualized health care to older patients. It is also recommended that all adults aged 65 years and over should be offered screening for frailty using a validated rapid frailty instrument suitable to the specific setting or context [33]. For this purpose, it is valuable to use valid and reliable frailty assessment instruments in daily practice that every healthcare professional can easily apply. In conclusion, our study supports the effectiveness of the use of a standardized approach to assess frailty. With this study, we have demonstrated that GFI is a valid and reliable scale in the Turkish older population.

### Electronic supplementary material

Below is the link to the electronic supplementary material.

**Supplementary Table 1**. The prevalence of Geriatric Syndromes according to GFI

## Data Availability

The data that support the findings of this study are available on request from the corresponding author, [M.G.]. The data are not publicly available due to. their containing information that could compromise the privacy of research participants.

## References

[1] Church S, Rogers E, Rockwood K, Theou O (2020). A scoping review of the clinical Frailty Scale. BMC Geriatr.

[2] Clegg A, Young J, Iliffe S, Rikkert MO, Rockwood K (2013). Frailty in elderly people. The Lancet.

[3] Cesari M, Calvani R, Marzetti E (2017). Frailty in older persons. Clin Geriatr Med.

[4] Morley JE, Vellas B, van Kan GA, Anker SD, Bauer JM, Bernabei R (2013). Frailty consensus: a call to action. J Am Med Dir Assoc.

[5] Mitnitski AB, Song X, Rockwood K (2004). The estimation of relative fitness and Frailty in Community-Dwelling older adults using self-Report Data. The Journals of Gerontology: Series A.

[6] Dent E, Kowal P, Hoogendijk EO (2016). Frailty measurement in research and clinical practice: a review. Eur J Intern Med.

[7] Steverink N, Slaets J, Schuurmans H, Lis M (2001). Measuring frailty: developing and testing the GFI (Groningen Frailty Indicator). Gerontologist.

[8] Metzelthin SF, Daniëls R, van Rossum E, de Witte L, van den Heuvel WJA, Kempen GIJM (2010). The psychometric properties of three self-report screening instruments for identifying frail older people in the community. BMC Public Health.

[9] Peters LL, Boter H, Buskens E, Slaets JP (2012). Measurement properties of the Groningen Frailty Indicator in home-dwelling and institutionalized elderly people. J Am Med Dir Assoc.

[10] Walston J, Buta B, Xue QL (2018). Frailty Screening and interventions: considerations for clinical practice. Clin Geriatr Med.

[11] Masnoon N, Shakib S, Kalisch-Ellett L, Caughey GE (2017). What is polypharmacy? A systematic review of definitions. BMC Geriatr.

[12] Podsiadlo D, Richardson S (1991). The timed up & go: a test of basic functional mobility for frail elderly persons. J Am Geriatr Soc.

[13] Katz S, Ford AB, Moskowitz RW, Jackson BA, Jaffe MW (1963). Studies of Illness in the aged: the index of ADL: a standardized measure of biological and psychosocial function. JAMA.

[14] Lawton MP, Brody EM (1969). Assessment of older people: self-maintaining and instrumental activities of daily living. Gerontologist.

[15] Rubenstein LZ, Harker JO, Salvà A, Guigoz Y, Vellas B (2001). Screening for undernutrition in geriatric practice: developing the short-form mini-nutritional assessment (MNA-SF). The Journals of Gerontology Series A: Biological Sciences and Medical Sciences.

[16] Durmaz B, Soysal P, Ellidokuz H, Isik AT (2018). Validity and reliability of geriatric depression scale-15 (short form) in Turkish older adults. North Clin Istanbul.

[17] Güngen C, Ertan T, Eker E, Yaşar R, Engin F (2002). Reliability and validity of the standardized Mini Mental State examination in the diagnosis of mild Dementia in Turkish population. Turk Psikiyatri Dergisi = Turkish Journal of Psychiatry.

[18] Cruz-Jentoft AJ, Bahat G, Bauer J, Boirie Y, Bruyère O, Cederholm T (2019). Sarcopenia: revised European consensus on definition and diagnosis. Age Ageing.

[19] van Abellan G, Rolland Y, Bergman H, Morley JE, Kritchevsky SB, Vellas B (2008). The I.A.N.A Task Force on frailty assessment of older people in clinical practice. J Nutr Health Aging.

[20] van Abellan G, Rolland Y, Andrieu S, Bauer J, Beauchet O, Bonnefoy M (2009). Gait speed at usual pace as a predictor of adverse outcomes in community-dwelling older people an International Academy on Nutrition and Aging (IANA) Task Force. J Nutr Health Aging.

[21] Flack VF, Afifi AA, Lachenbruch PA, Schouten HJA (1988). Sample size determinations for the two rater kappa statistic. Psychometrika.

[22] Hymabaccus Muradi BAB. Yaşlılarda Kırılganlığı Ölçmeye Yönelik FRAİL Ölçeğinin Türkçe Geçerlik ve Güvenirlik Çalışması. 2017.

[23] Xiang W, Cheng Y, Li Z, Han J, Li K (2020). Cross-cultural adaptation and validation of the Groningen Frailty Indicator in Chinese nursing home residents. Aging Clin Exp Res.

[24] Aygör H, Sahin S, Senuzun Aykar F, Fadiloglu Ç, Akçiçek F. P131: testing the reliablity and validity of the ‘Groningen Frailty Index’ in Turkish population. Eur Geriatr Med. 2014;5.

[25] Braun T, Gruneberg C, Thiel C (2018). German translation, cross-cultural adaptation and diagnostic test accuracy of three frailty screening tools: PRISMA-7, FRAIL scale and Groningen Frailty Indicator. Z Gerontol Geriatr.

[26] Tian X, Qiao X, Dong L, Liu N, Si H, Jin Y (2020). Cross-cultural adaptation and psychometric properties of the Groningen Frailty Indicator (GFI) among Chinese community-dwelling older adults. Geriatr Nurs.

[27] Olaroiu M, Ghinescu M, Naumov V, Brinza I, Heuvel W (2014). The psychometric qualities of the Groningen Frailty Indicator in Romanian community-dwelling old citizens. Fam Pract.

[28] O’Caoimh R, Sezgin D, O’Donovan MR, Molloy DW, Clegg A, Rockwood K (2020). Prevalence of frailty in 62 countries across the world: a systematic review and meta-analysis of population-level studies. Age Ageing.

[29] Vermeiren S, Vella-Azzopardi R, Beckwee D, Habbig AK, Scafoglieri A, Jansen B (2016). Frailty and the prediction of negative Health outcomes: a Meta-analysis. J Am Med Dir Assoc.

[30] Hoogendijk EO, Afilalo J, Ensrud KE, Kowal P, Onder G, Fried LP (2019). Frailty: implications for clinical practice and public health. The Lancet.

[31] Daniels R, van Rossum E, Beurskens A, van den Heuvel W, de Witte L (2012). The predictive validity of three self-report screening instruments for identifying frail older people in the community. BMC Public Health.

[32] Bongue B, Buisson A, Dupre C, Beland F, Gonthier R, Crawford-Achour É (2017). Predictive performance of four frailty screening tools in community-dwelling elderly. BMC Geriatr.

[33] Dent E, Morley JE, Cruz-Jentoft AJ, Woodhouse L, Rodriguez-Manas L, Fried LP (2019). Physical Frailty: ICFSR International Clinical Practice guidelines for Identification and Management. J Nutr Health Aging.

