# Supplementary material for: Turkish translation, cross-cultural adaptation and reliability of the Groningen Frailty Indicator
Source: BMC Geriatr. 2023 Nov 17;23:753. doi: 10.1186/s12877-023-04445-5 (PMC10656956; doi:10.1186/s12877-023-04445-5)
Supplement: Supplementary file 1 — Supplementary Table 1. The prevalence of Geriatric Syndromes according to GFI [file 12877_2023_4445_MOESM1_ESM.docx]

**Supplementary Table 1.The prevalence of Geriatric Syndromes according to GFI**

|  | **Robust (N=34)**  **N,%** | **Frail (N=66)**  **N,%** | **P** |
| --- | --- | --- | --- |
| **Multimorbidity ≥2** | 20 (58.8) | 49 (74.2) | 0.11 |
| **Urinary Incontinence** | 8 (23.5) | 34 (50.7) | 0.01 |
| **History of Falls** | 4 (11.8) | 18 (26.9) | 0.08 |
| **Polypharmacy** | 13 (38.2) | 40 (59.7) | 0.04 |
| **Drug number** | 3.5 (3.0) | 6.0 (5.0) | 0.01 |
| **Katz Index of Independence in Activities of Daily Living** | 6.0 (0.0) | 6.0 (1.0) | 0.01 |
| **Lawton-Brody Instrumental Activities of Daily Living Scale** | 8.0 (0.0) | 8.0 (2.0) | 0.001 |
| **Mini Nutritional Assessment-Short Form** | 14.0 (1.3) | 13.0 (5.0) | 0.001 |
| **Mini-mental State Exam** | 29.0 (3.0) | 27.0 (6.0) | 0.001 |
| **Yesavage Geriatric Depression Scale-15** | 0.0 (2.0) | 4.0 (3.0) | <0.001 |
| **SARC-F** | 0.0 (1.0) | 1.0 (3.0) | <0.001 |
| **Grip strength (kg)** | 24.84±7.48 | 19.62±7.06 | 0.001 |
| **Gait speed (m/sec)** | 1.05±0.31 | 0.88±0.36 | 0.02 |
